# Realizing Synchronous Energy Harvesting and Ion Separation with Graphene Oxide Membranes

**DOI:** 10.1038/srep05528

**Published:** 2014-07-02

**Authors:** Pengzhan Sun, Feng Zheng, Miao Zhu, Kunlin Wang, Minlin Zhong, Dehai Wu, Hongwei Zhu

**Affiliations:** 1 School of Materials Science and Engineering, State Key Laboratory of New Ceramics and Fine Processing, Key Laboratory of Materials Processing Technology of MOE, Tsinghua University, Beijing 100084, P. R. China; 2 Center for Nano and Micro Mechanics, Tsinghua University, Beijing 100084, P. R. China; 3 Department of Mechanical Engineering, Tsinghua University, Beijing 100084, P. R. China

## Abstract

A synchronous ion separation and electricity generation process has been developed using G-O membranes. In addition to the size effect proposed prevsiouly, the separation of ions can be attributed to the different interactions between ions and G-O membranes; the generation of electricity is due to the confinement of G-O membranes, and the mobility difference of ions. Efficient energy transduction has been achieved with G-O membranes, converting magnetic, thermal and osmotic energy to electricity, distinguishing this material from other commercial semi-permeable membranes. Our study indicated that G-O membranes could find potential applications in the purification of wastewater, while producing electricity simultaneously. With G-O membranes, industrial magnetic leakage and waste heat could also be used to produce electricity, affording a superior approach for energy recovery.

In biological systems, ion-selectivity can be achieved by the delicate interactions between ions and amino acids^1^,^2^,^3^,^4^,^5^,^6^. Specifically, proteins primarily utilize two types of interactions to recognize different ions: the coordination between transition metal cations and oxygen-containing functional groups^7^,^8^,^9^,^10^, and the non-covalent cation-π interactions between main group cations and aromatic side chains^11^,^12^,^13^,^14^. The function of ion-channel proteins, however, could be mimicked by nano-structured materials like graphene oxide (G-O) membranes to afford excellent ion selectivity^15^,^16^,^17^,^18^. In a typical G-O sheet, oxygen functionalities asymmetrically cluster on the basal plane and the edges, resulting in the formation of small *sp*^*2*^ clusters within the *sp*^*3*^ C-O matrix^19^,^20^,^21^. When G-O sheets are stacked together, the hydrogen bonding between flakes can strongly hold them together to establish a laminate structure, which should possess adequate mechanical strength suitable for further manipulations^15^,^16^,^17^,^18^. On the other hand, a nano-capillary network is also evident in the layered G-O structure^15^,^16^,^17^,^18^,^22^,^23^,^24^, which enables water molecules to permeate the membranes with an abnormally low friction while separates different ions simultaneously. Consequently, these remarkable properties have made G-O membranes promising barrier materials for a diverse set of solutions. Furthermore, energy conversion in G-O membranes is expected to be highly efficient, due to the confinement of G-O nano-capillaries at nano-scale^25^,^26^,^27^. Here we report an ion separation process accompanied with synchronous electricity generation with G-O membrane filters (Figure 1a). The theoretical hypothesis of our discovery is that transition metal cations prefer to bind to the *sp*^*3*^ matrix of G-O sheets via a coordination interaction, whereas cations lacking *d* electrons could bind to *sp*^*2*^ clusters via a cation-π interaction^17^,^18^, as shown in Figure 1b. Therefore, we envisioned that G-O membranes could find broad applications in both wastewater treatment and sustainable energy generation, with appropriate large-scale integration (Figure 1c).

## Results

### Characterizations of G-O membranes

**Figure 2:**
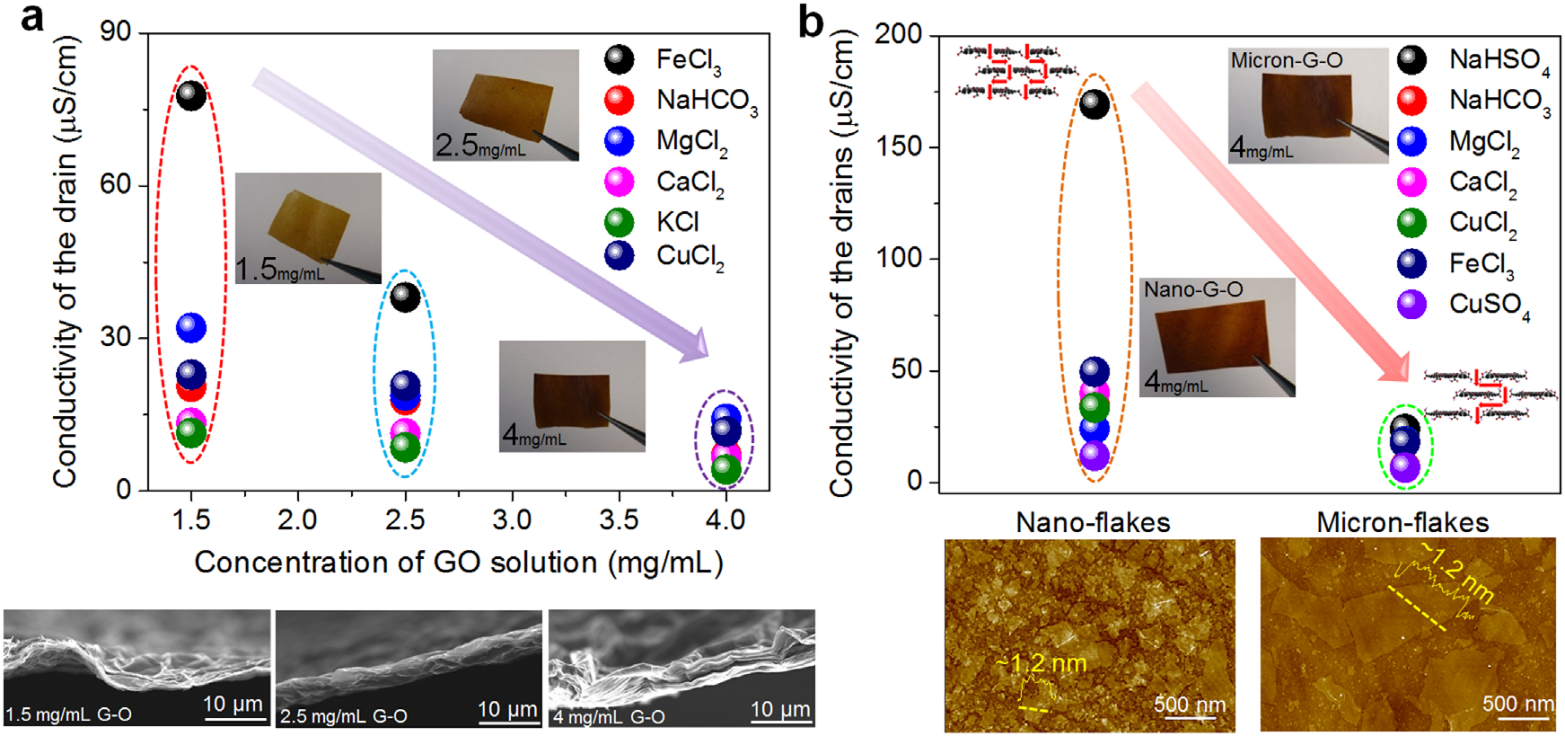
Characterizations of the ion transport through G-O membranes with different features. (a), Conductivities of the drains after 3 h for considered sources through G-O membranes. Bottom panel shows corresponding cross-section SEM characterizations of as-prepared G-O membranes drop-casted by 1.5, 2.5 and 4 mg/mL. The insets show the as-assembled G-O membranes. (b), Conductivities of the drains after 3 h for considered sources through G-O membranes drop-casted from nanosize and microsize G-O sheets, respectively. The insets show the photographs of corresponding G-O membranes and the schematic drawings for nanocapillaries formation. Bottom panel shows AFM characterizations of as-prepared microsize and nanosize G-O flakes. The insets show the corresponding height profiles of G-O sheets. For each group of experiments, at least three runs were repeated. Excellent reproducibility could be obtained and the variations were within the size of the data ball.

Utilizing G-O membrane, we aim to develop a novel ion separation process accompanied with synchronous energy production. Particularly, G-O membranes featuring different physicochemical properties were allowed to undergo penetration process with selected ions (see Methods and Materials Section in Supplementary Information for detailed penetration experiments), as illustrated in Figure 2. We first examined the effect of membrane thickness, and a series of G-O membranes with increasing thicknesses have been readily prepared by drop-casting G-O solutions with 1.5, 2.5 and 4 mg/mL concentrations, as indicated in the insets in Figure 2a. At the same time, it appears that increasing the thickness of G-O membranes could effectively inhibit the permeation of source ions (Figure 2a, Figure S2). Additionally, SEM analysis of the cross-sections has indicated that membrane thicknesses increased with the concentration of G-O preparation solutions, and a lamellar structure was generally obtained. Notably, the thickness of G-O membranes prepared was less than 10 μm, and the thinnest membrane was ~1 μm thick, which was prepared from the 1.5 mg/mL G-O preparation solution.

Drop-casting G-O sources should lead to the formation of freestanding laminates, in which the clustered oxygen-containing functional groups on G-O sheets tended to depart the empty and non-oxidized regions to form a nano-capillary network^15^,^28^. The quantity of such structure, however, is dependent on the lateral size of G-O sheets. On the other hand, the lateral dimension of G-O flakes could also alter the penetration behavior of certain ions, thus micron- and nano-sized G-O flakes have been synthesized by the modified Hummers' method, starting from natural and worm-like graphite^29^,^30^. Subsequently, those flakes were characterized by atomic force microscopy (AFM) analyses, and the results are illustrated in Figure 2b. To our delight, it appears that the G-O sheets prepared possessed a monolayer structure, as indicated in the insets of Figure 2b. G-O membranes composed of micron- or nano-sized flakes have been prepared by drop-casting the 4 mg/mL G-O source solution. Plus, the penetration performances of different salts have also been investigated, and the results are shown in Figure 2b as well. Apparently, the penetration of certain ion sources could be significantly enhanced by using G-O sheets with nano-scaled lateral dimensions, which can be attributed to the increase of G-O nano-capillaries (Figure 2b, Figure S3). The presence of functional groups on G-O surfaces could considerably change the configuration of nano-capillaries and the interlayer distances, thus we next examined the ion penetration processes through G-O membranes at different degree of reduction, as shown in Figure S4. Interestingly, the permeability of selected ions declined with the addition of reducing reagents. All of the results described above have clearly demonstrated that the ion penetration processes of G-O membranes could be effectively modulated by controlling a number of factors, such as the thicknesses, the reduction degrees of G-O membranes, and the lateral dimensions of G-O flakes. These results have not only warranted further studies on ion separation, but also initiated investigations to utilize G-O membranes in energy generation.

### Ion separation

Initially, ions were allowed to permeate the thinnest G-O membrane for 3 h, and the filtrates were collected and subject to emission spectroscopy analyses, affording accurate concentrations for cations, as summarized in Figure S5. Using Cl^−^ as the counter ion, the permeability of various cations appears to be in the order of Mg^2+^, Na^+^ > Cd^2+^ > Ba^2+^ > Ca^2+^ > K^+^ > Cu^2+^ > Fe^3+^. This result suggested that in addition to size effect proposed by Joshi, et al.^16^, the diverse interactions between ions and G-O sheets also contributed to the selectivity of G-O membranes. In detail, the cation-π interactions, present between alkali or alkaline earth cations and the *sp*^*2*^ clusters of G-O nano-capillaries, should be weaker than the coordination interactions between transition metal ions and the *sp*^*3*^ matrix of G-O sheets. However, the coordination of a soft metal cation Cd^2+^ seemed to be weaker than Cu^2+^ and Fe^3+^, possibly due to the large distance between Cd^2+^ and the oxygen-containing functional groups^17^. In addition, it appears that the concentrations of a cation could also be affected by its source. For instance, the concentrations of Cu^2+^ and Cd^2+^ originated from CuCl_2_ and CdCl_2_ were both higher than CuSO_4_ and CdSO_4_. This finding indicated that the through-membrane transport of a cation could be controlled by the electrostatic attraction from its counter anion. As illustrated in Figure S6, the pH values of the drains from various sources, such as NaHSO_4_, NaHCO_3_, CuCl_2_, CuSO_4_ and FeCl_3_, have been efficiently determined. After ion penetration, the filtrates from NaHSO_4_ and FeCl_3_ sources appeared to be strongly acidic; the filtrate from NaHCO_3_ source was basic; the filtrates from CuCl_2_ and CuSO_4_ sources, however, turned out to be weakly acidic. With the pH values of NaHSO_4_ and FeCl_3_, we could easily calculate the H^+^ concentrations in drains by the following equation: pH = -lg*c_H+_*. As expected, the permeability of H^+^ was superior to metal ions employed in our study.

### Energy harvesting

**Figure 3:**
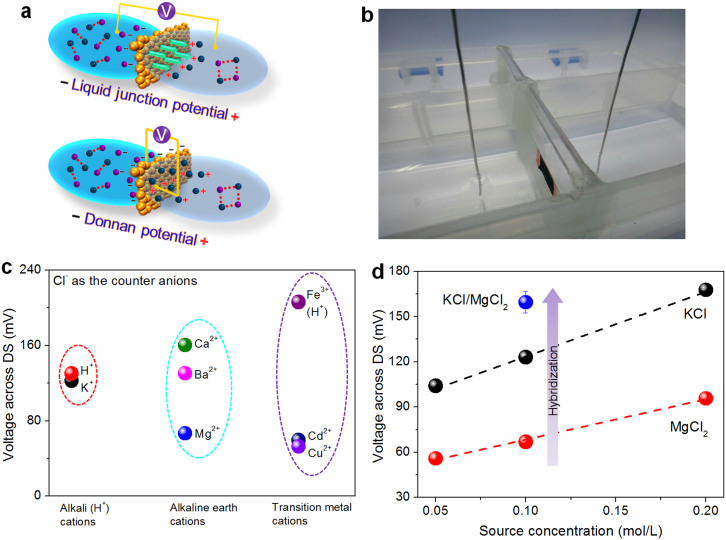
Voltage generations based on ion transport through G-O membranes. (a),(b), Schematic diagrams and photograph of the voltage measuring process. (c), Stable voltage generations based on the penetrations of alkali, alkaline earth, and transition metal salts through G-O membranes for 3 h. (d), Generated voltages during the transport processes of KCl, and MgCl_2_ with increasing concentrations. The blue ball plots the generated voltage for the hybrid of KCl, and MgCl_2_ penetrating through G-O membranes for 3 h.

After membrane separation, the ions in sources and drains possessed different chemical potentials. In this case, ions would be spurred by a diffusion driving force that advanced them to the drains. For ions passing through G-O membranes, they possessed different diffusivities, due to the different interactions between ions and G-O nano-capillaries, resulting in difference on ionic mobility. Consequently, such mobility difference would lead to the presence of an excess amount of cations or anions in both drains and sources, which in turn would produce a trans-membrane electric potential Δ*V*_DS_, as illustrated in Figure 3a (top panel).

Based on the electric potentials of drains (*V*_D_) and sources (*V*_S_), the electric potential difference between the drains and sources (Δ*V*_DS_ = *V*_D_-*V*_S_) during the ion transporting processes has been determined, as illustrated in Figure 3a,b. For the penetration of chloride salts, we have found that the electric potential of drains was higher than the sources, affording Δ*V*_DS_ > 0, as shown in Figure 3c. In aqueous environment, however, due to the ionization of the functional groups presiding on G-O sheets, G-O membranes were negatively charged, which would give rise to significant repulsion forces between membranes and Cl^−^ anions. Consequently, the penetration of Cl^−^ would be prohibited and such process was predominately driven by the attracting force between positively charged cations and negatively charged anions. In contrast, cations were attracted by the G-O nano-capillaries, and would permeate through G-O membranes promptly. Therefore, cations and anions in both drains and sources should be effectively enriched, resulting in a higher electric potential in the drains (top panel of Figure 3a). In addition, substantial difference has been observed in the Δ*V*_DS_ values of chlorides, which could be originated from the mobility difference of various ions, as shown in Figure 3c. During the course of ion transporting through G-O membranes, a greater mobility difference for either cations or anions often leads to a larger Δ*V*_DS_.

We next examined the effect of source concentration on Δ*V*_DS_, as illustrated in Figure 3d. It appears that increasing the concentration of the source solutions could adequately increase the voltage generated. Notably, hybridizing two different sources have afforded a larger Δ*V*_DS_, comparing with the ones obtained from single-component sources (Figure 3d). At the same time, several sulfates have been allowed to penetrate through G-O membranes, and the corresponding Δ*V*_DS_ were also determined (Figure S7), suggesting that the voltages generated by sulfates were smaller than chlorides. Therefore, we concluded that hybridizing approach or using highly-concentrated chloride solutions in G-O membranes would be advantageously preferred for electricity production.

We next investigated the possibility of generating electric potential difference across G-O membranes, which could be obtained from the following equation, Δ*V*_GDS_ = *V*_GD_-*V*_GS_. In this equation, *V*_GD_ and *V*_GS_ are the electric potentials of the G-O surfaces that have direct contact with the drain and source solutions, and Δ*V*_GDS_ serves the same roles as Δ*V*_DS_. Indeed, a voltage across G-O membrane has been observed and measured during the penetration process, although it seemed to be smaller than Δ*V*_DS_, as shown in Figure S8. In this study, the separation of sources and drains by G-O membranes has effectively prevented the free transportation of source ions, resulting in the establishment of an electrochemical dialysis equilibrium, which consequently should generate a Donnan potential, as illustrated in Figure 3a (bottom panel). Theoretically, the trans-membrane diffusion of ions would lead to the formation of a space charge zone. The negatively charged G-O membranes repulsed anions and attracted cations, thus cations would be the first ones transported into membranes through G-O nano-capillaries, interacting with G-O sheets. Driven by the attraction force from cations embedded in G-O membranes, anions subsequently underwent penetration processes. Consequently, excessive cations would aggregate on the G-O surfaces that directly contact with drain solutions, whereas excessive anions would aggregate on the G-O surfaces that directly contact source solutions, which ultimately led to the generation of a Donnan potential (bottom panel of Figure 3a).

**Figure 4:**
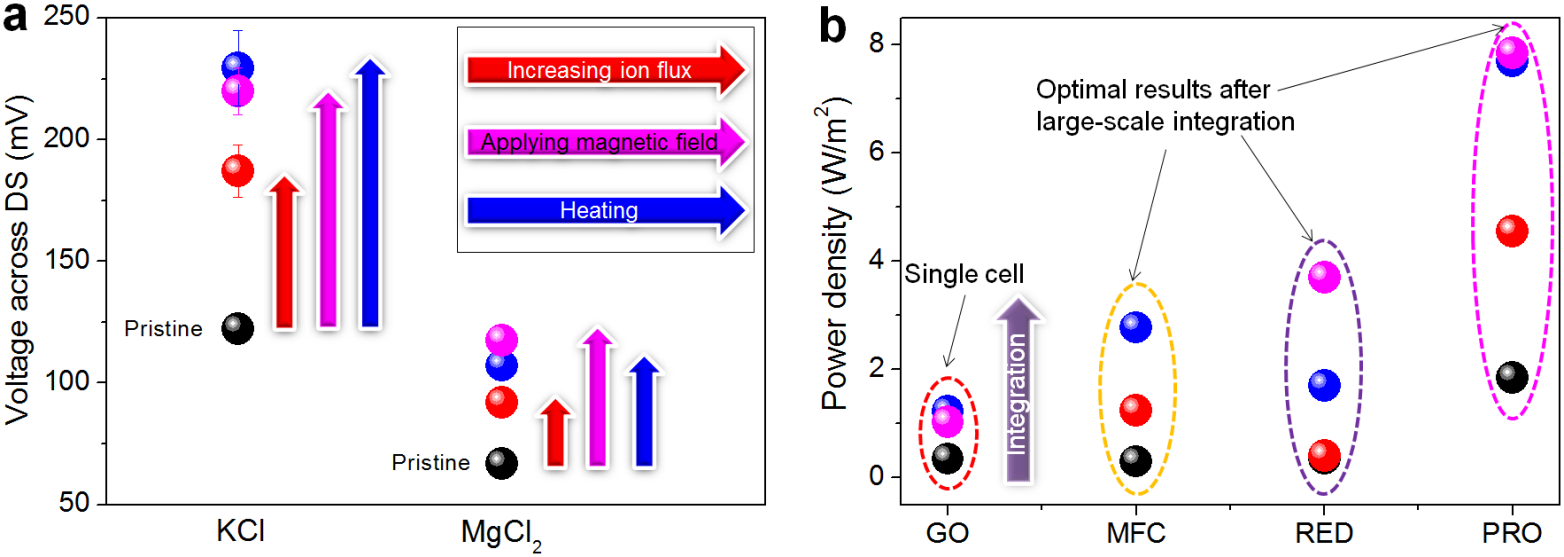
Energy transductions involved in the ion trans-membrane transport processes. (a), Voltage generations Δ*V*_DS_ across drains and sources with external stimuli (*e.g.* increasing ion flux, applying magnetic fields and heating). (b), Comparison of generated power densities based on ion separations with G-O membrane, PRO, RED and MFC. The data for PRO, RED and MFC were cited from Ref. 21.

Specially, the possible energy transduction involved in the ion trans-membrane transport processes was studied. In an effort to understand the effect of diffusive area, the electric potential difference Δ*V*_DS_ across G-O membranes with different diffusive areas was further investigated using 0.1 mol/L KCl and MgCl_2_ source solutions, as illustrated in Figure 4a. Notably, when the diffusive area of a G-O membrane was reduced from 19.6 to 7.1 mm^2^, a significant increase was evident in Δ*V*_DS_. Presumably, reducing the diffusive area of a membrane should increase its ion flux, the ion permeation rate per unit area, thus the corresponding confinement effect and osmotic energy conversion would be enhanced^25^, resulting in more cations and anions enriched in drains and sources respectively and further the increase of Δ*V*_DS_. We next examined the voltage generations of G-O membranes under external magnetic fields. Specifically, both perpendicular (~10 mT) and parallel (~30 mT) magnetic fields have been applied to G-O membranes and the results were illustrated in Figure 4a. Suprisingly, it appears that the corresponding Δ*V*_DS_ generated were significantly higher under magnetic conditions, indicating a possible energy transduction process converting magnetism to electricity. Encouraged by these results, the electric potential difference Δ*V*_DS_ for a G-O membrane has also been closely investigated at different temperatures, as shown in Figure 4a. When temperature was increased from 20 to 40°C, the values of measured Δ*V*_DS_ significantly increased, which certainly demonstrated an effective transduction from thermal energy to electricity (Detailed voltage generations across drains and sources with external simuli were analyzed and shown in Figures S9–S12).

## Discussion

The generated power densities based on G-O membranes were compared with the optimized cases of existing membrane-based energy generation processes such as PRO (pressure-retarded osmosis), RED (reverse electrodialysis) and MFC (microbial fuel-cell)^27^, as shown in Figure 4b. It reveals that the pristine power densities generated by G-O membranes without integration are smaller than the optimized large-scale integrated results of PRO, RED and MFC. However, when applying magnetic fields or heating, the generated power densities based on single G-O membrane can be enhanced significantly and lie in the same order of magnitude as the best cases of PRO, RED and MFC. Therefore, it is reasonable to deduce that after integration on a large scale, the performance of energy generation based on ion permeating G-O membranes under the conditions of collecting magnetism or heat can be improved markedly, which may exceed the existing membrane-based energy production technologies (Studies on the practical energy harvesting ability of G-O membranes were included in Supplementary Information, Table S1, S2 and Figures S13–S14).

When different ions were allowed to penetrate through G-O membranes, transition metal cations prefer to coordinate to the *sp*^*3*^ C-O matrix of G-O sheets, whereas cations lacking *d* electrons would bind to *sp*^*2*^ clusters through cation-π interactions. On the other hand, anions would confront a strong repulsion force, due to the fact that G-O membranes were negatively charged. Consequently, such difference in mutual interactions has enabled effective ion separation processes in G-O membranes, beyond the size effect proposed by Joshi, et al.^16^ Particularly, electrical voltages can be generated across drains and sources (Δ*V*_DS_), or between the contacting surfaces of G-O membranes (Δ*V*_GDS_). In addition, several types of energy such as magnetism, thermal and osmotic energy, have been effectively converted to electricity using G-O membranes (Amazingly, the power densities generated by G-O membranes under the conditions of heating or magnetic fields were increased to >300%, see Figure S12 and Table S2), which was not achievable by other semi-permeable membranes (The inherent ion separation, energy generation and conversion properties of G-O membranes were proved by control experiments using commercial microfilters, as shown in Figures S15–S17). Therefore, we have proved that G-O membranes could afford a synchronous ion separation and energy generation process, due to their unique structural properties (Studies on the selective penetrations and voltage generations of hybrid sources were shown in Figure S18). To our delight, we also found that hybridization of source solutions have improved potential difference Δ*V*_DS_, indicating that industrial wastewater, brine solutions and seawater could also serve as source solutions for the electricity production using G-O membranes. Furthermore, during the ion penetration process, G-O membranes could undergo efficient energy transduction, converting magnetic and thermal energy to electricity, thus, industrial magnetic leakage and waste heat could be recovered to generate electricity. In conclusion, we have demonstrated that G-O membranes were superior material for ion separation and energy transduction, and they could find latent industrial applications in synchronous waste water purification and energy generation.

## Author Contributions

H.W.Z. and P.Z.S. conceived and designed the experiments. P.Z.S., F.Z. performed the experiments. M.Z. and M.L.Z. conducted the theoretical analysis. K.L.W. and D.H.W. interpreted the ion separation results. P.Z.S. and H.W.Z. co-wrote the manuscript.

## Supplementary Material

Supplementary Information SUPPLEMENTARY INFO

## Figures and Tables

**Figure 1:**
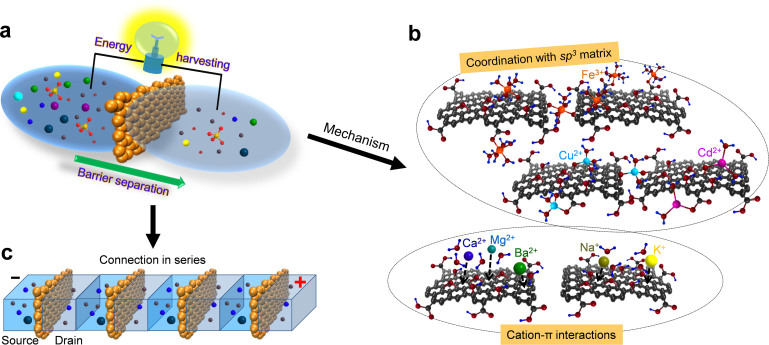
Schematics of the ion transport processes. (a), Schematic diagram of the synchronous ion separation and energy generation process with G-O membranes. (b), The proposed mechanism: the noncovalent cation-π interactions of alkali and alkaline earth cations with *sp*^2^-clusters and the coordination between transition metal ions and *sp*^3^ matrix. (c), The integration of the penetration systems for practical applications.
