# Supplementary material for: Realizing Synchronous Energy Harvesting and Ion Separation with Graphene Oxide Membranes
Source: Sci Rep. 2014 Jul 2;4:5528. doi: 10.1038/srep05528 (PMC4078314; doi:10.1038/srep05528)
Supplement: Supplementary Information — SUPPLEMENTARY INFO [file srep05528-s1.doc]

**Supplementary Information**

Realizing Synchronous Energy Harvesting and Ion Separation with Graphene Oxide Membranes

Pengzhan Sun, Feng Zheng, Miao Zhu, Kunlin Wang, Minlin Zhong, Dehai Wu, Hongwei Zhu

Content

[Methods and Materials 2](#__RefHeading___Toc381018916)

[Figure S1. Photograph of the home-made permeation apparatus. 3](#__RefHeading___Toc381018917)

[Figure S2. Penetrations of selected salt solutions through G-O membranes prepared by drop-casting G-O solutions with increasing concentrations. 4](#__RefHeading___Toc381018918)

[Figure S3. Penetrations of selected sources through G-O membranes prepared from nano- or micron-sized G-O flakes. 4](#__RefHeading___Toc381018919)

[Figure S4. Penetrations of selected salt solutions through G-O membranes chemically reduced for varied degrees. 5](#__RefHeading___Toc381018920)

[Figure S5. Cation concentrations for the filtrates in drains of selected salt solutions after 3h penetration. 6](#__RefHeading___Toc381018921)

[Figure S6. pH variations of the filtrates in drains of selected salt solutions during the penetration process. 8](#__RefHeading___Toc381018922)

[Detailed principle for the generation of the trans-membrane electrical potential Δ*V*_DS_ across G-O membranes (illustrated in Figure 3a). 8](#__RefHeading___Toc381018923)

[Figure S7. Voltage generations across sources and drains Δ*V*_DS_ based on chlorides and sulphates with the same cations. 10](#__RefHeading___Toc381018924)

[Figure S8. Voltage generations across G-O membranes Δ*V*_GDS_ during the penetration process. 10](#__RefHeading___Toc381018925)

[The detailed principle for the generation of Donnan potential Δ*V*_GDS_ across G-O membranes (illustrated in Figure 3a). 10](#__RefHeading___Toc381018926)

[Figure S9. Voltage generations Δ*V*_DS_ across G-O membranes with different diffusive areas. 11](#__RefHeading___Toc381018927)

[Figure S10. Penetrations of selected salts and voltage generations across sources and drains when magnetic fields are applied. 12](#__RefHeading___Toc381018928)

[Figure S11. Voltage generations across sources and drains at different temperatures. 13](#__RefHeading___Toc381018929)

[Figure S12. Comparison of voltage generations across drains and sources under the conditions of heating and applying magnetic field. 14](#__RefHeading___Toc381018930)

[Table S1. Practical energy generations based on G-O membranes. 14](#__RefHeading___Toc381018931)

[Table S2. Energy generations based on G-O membranes under the conditions of heating and applying magnetic field. 15](#__RefHeading___Toc381018932)

[Figure S13. Practical energy harvesting based on the penetrations of 1M/0.01M KCl through G-O membranes in series. 17](#__RefHeading___Toc381018933)

[Figure S14. Photograph of the penetrating system in series based on the penetration of FeCl_3_ through G-O membranes. 17](#__RefHeading___Toc381018934)

[Figure S15. Transport properties based on commercial microfilters (220 nm). 19](#__RefHeading___Toc381018935)

[Figure S16. Transport properties based on commercial microfilters (220 nm). 20](#__RefHeading___Toc381018936)

[Figure S17. Transport properties based on commercial microfilters (220 nm). 20](#__RefHeading___Toc381018937)

[Figure S18. Synchronous ion separation and energy generation based on G-O membranes. 21](#__RefHeading___Toc381018938)

[References 22](#__RefHeading___Toc381018939)

## Methods and Materials

***Preparation of G-O flakes and freestanding G-O membranes.*** Micron- and nano-sized G-O flakes were prepared by the modified Hummers’ method using natural graphite (1) and worm-like graphite (2), respectively. Based on these two kinds of G-O sheets, the freestanding G-O membranes were formed by drop-casting onto a piece of smooth paper. After dried thoroughly, the as-prepared membranes are peeled off and stored for penetration experiments.

***Chemical reduction of G-O membranes.*** As-prepared G-O membranes were reduced at 60^o^C through a chemical route using hydrazine vapor as the reduction agent. All the chemical reduction processes were performed with membranes drop-casted by 2.5 mg/mL G-O solutions.

***Characterizations.*** As-prepared G-O flakes and corresponding G-O membranes were characterized by scanning electron microscope (SEM, LEO 1530, 10kV), atomic force microscopy (AFM, Agilent 5100), X-ray Diffraction (XRD, Siemens, 08DISCOVER, λ=0.15405 nm) and X-ray Photoelectron Spectroscopy (XPS, PHI Quantera SXM, AlKα).

***Penetration experiments.*** The penetration experiments were conducted with a home-made apparatus (**Figure S1**). Briefly, the source and drain were separated by a plastic plate with a hole (5 mm in diameter) in the center. G-O membranes were sealed onto this hole by double faced copper tapes with an aperture (5 mm and 3 mm in diameter) in the same position to ensure the G-O membrane directly contact the solutions in sources and drains. 80 mL of 0.1 mol/L certain source solution and deionized water were injected into the sources and drains with the same speed. During the penetration process, the conductivities of the drains were measured by a conductivity meter (INESA, DDS-307) and the pH values were measured by a pH meter (SANXIN, MP523) under a mild mechanical stirring. Before the permeation experiments, the excellent seal of double faced copper tapes was confirmed by sealing the entire hole with a piece of double faced copper tape and measuring the conductivity variations of the drains with time when the source vessel was injected with 80 mL 0.1 mol/L certain electrolyte. We found that no obvious conductivity variations occurred with time in the drain solutions, demonstrating the excellent seal of double sized copper tapes used in this work.

***Electricity generation measurements.*** During the penetration process, the voltage across sources and drains (Δ*V*_DS_=*V*_D_-*V*_S_) was measured by a pair of identical silver electrodes (99.99% in purity) separated by the G-O membrane in the middle with a total distance of 4 cm. The silver electrodes were fixed on a settled electrode stand. The voltage generation across G-O membrane (Δ*V*_GDS_=*V*_GD_-*V*_GS_) was measured by making two electrodes with two double faced copper tapes on both surfaces of the G-O membrane. The voltages were measured by Keithley 2601. Unless otherwise specified the G-O membranes used for ion separation and electricity generation experiments were drop-casted by 1.5 mg/mL G-O sources (with the thinnest thickness).


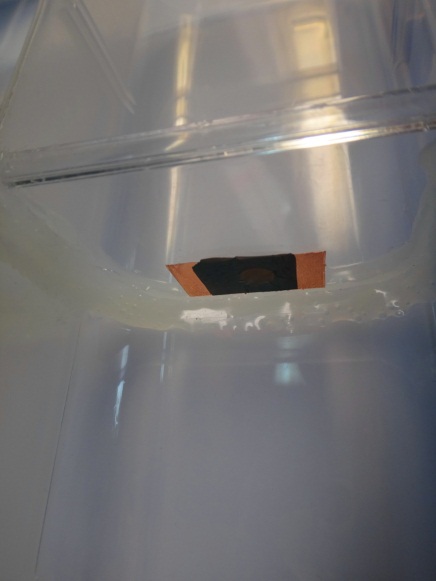


## Figure S1. Photograph of the home-made permeation apparatus.

## Figure S2. Penetrations of selected salt solutions through G-O membranes prepared by drop-casting G-O solutions with increasing concentrations.

**(a)** 1.5 mg/mL, **(b)** 2.5 mg/mL, **(c)** 4 mg/mL. The solid lines are guides to eyes.

## Figure S3. Penetrations of selected sources through G-O membranes prepared from nano- or micron-sized G-O flakes.

SEM characterizations of **(a)** nano- and **(b)** micron-sized G-O flakes, respectively. Ion penetrations through G-O membranes composed of **(c)** nano- and **(d)** micron-sized G-O flakes, respectively. The solid lines are guides to eyes.

As shown in Fig. S3a, during the initial 1 h of permeation, the drain conductivities only increase slightly within a small range, which can be attributed to the gradual wetting procedure of the GO membranes in aqueous environment. Afterwards, the conductivities of the drain solutions increase rapidly with time, which can be attributed to the transport of ions by the fast water flows within the GO membranes.

The complete path of hydrated ions through a GO membrane with a thickness of *h* involves a number of turns *N=h/d* (*d* is the interlayer distance within the GO membrane), each turn involves a capillary length *L* (here *L* is the lateral size of the GO sheets within the GO membrane). Therefore, the total number of the channels per unit area can be calculated by *N×L/L^2^=h/Ld*, indicating that the total number of the channels per unit area is in inverse proportion to the lateral size of the GO sheets. As the average lateral size *L* decreases, the total number of nanocapillaries increases, leading to the acceleration of the ion permeation through GO membranes.

## Figure S4. Penetrations of selected salt solutions through G-O membranes chemically reduced for varied degrees.

**(a-d)** XPS spectra of G-O membranes chemically reduced for 0 h, 1 h, 3 h and 5 h, respectively. **(e-h)** Corresponding conductivities of the drains for selected salt solutions through G-O membranes reduced for varied degrees. The solid lines are guides to eyes.

The results indicate that with the chemical reduction of GO membranes, the ion trans-membrane permeations are weakened gradually, which can be attributed to the gradual decrease of the amount of oxygen containing functional groups and further the decrease of the interlayer distances between GO layers within the membranes.

## Figure S5. Cation concentrations for the filtrates in drains of selected salt solutions after 3h penetration.

The H^+^ concentrations of the filtrates for NaHSO_4_ and FeCl_3_ can be inferred from the corresponding pH values (pH = -lg*c_H+_*), as shown in **Figure S6**. From the above results, it can be concluded that the permeabilities of the selected cations are in the order of Mg^2+^, Na^+^ > Cd^2+^ > Ba^2+^ > Ca^2+^ > K^+^ > Cu^2+^ > Fe^3+^ with the counter Cl^-^ anions, while protons exhibit an anomalous faster transport rate. These results indicate that the coordination interactions between transition metal ions and the *sp^3^* clusters are stronger than the cation-π interactions of alkali and alkaline earth cations with the π networks within the G-O nanocapillaries with an exception of the soft metal ion Cd^2+^, in which case the coordination interaction with the *sp^3^* matrix is much weaker than the cases in Cu^2+^ and Fe^3+^.

Particularly, in the case of alkali and alkaline earth cations, if no water molecules are present within the G-O nanocapillaries, the binding energies between the considered metal cations and graphene follow the order of K > Ba > Na > Ca > Cu > Cd according to the simulation results obtained by first-principles calculations based on the plane-wave-basis-set density functional theory (DFT) approach (3). These simulation results indicate that the cation-π interactions of main group cations with the *sp^2^* clusters of G-O membranes are significantly stronger than those of transition metal cations, further confirming that the main group cations mainly bind to the *sp^2^* regions while the transition metal ions prefer to coordinate to the *sp^3^* matrix of G-O membranes. However, in aqueous environment, due to the shielding effect of the hydration shell, the strengths of cation-π interactions are reordered (4, 5) and the balance between binding interactions and desolvation effect of cations are responsible for the selectivity of G-O membranes. In detail, due to the higher hydration energies of the smaller Mg^2+^ and Na^+^, the first hydration shells of Mg^2+^ and Na^+^ ions within the G-O nanocapillaries tend to remain intact (4, 5, 6), while the cation-π bindings of the larger Ba^2+^, Ca^2+^ and K^+^ are sufficiently strong to result in partial desolvation of the cations and the *sp^2^* clusters of G-O sheets will substitute some of the water molecules to directly contact with the cations (6, 7). Therefore, the cation-π bindings of Ba^2+^, Ca^2+^ and K^+^ are preferred over Mg^2+^ and Na^+^ ions, leading to the greater penetrations of Mg^2+^ and Na^+^ than those of Ba^2+^, Ca^2+^ and K^+^.

In the case of Cu^2+^ and Cd^2+^, Cu^2+^ ions favor square-planar coordination geometry (8), while the soft-metal Cd^2+^ ions favor linear or tetrahedral coordination (8, 9). In terms of binding to oxygen-containing functional groups (e.g. carboxylate groups), Cd^2+^ ions prefer to bind in a direct confirmation, where two carboxylate oxygen atoms are shared equally, whereas the Cu^2+^ ions tend to bind in a *syn* confirmation (the ion is on the same side of C-O bond as the C=O), which is more stable (10). These differences result in a much smaller distance between Cu^2+^ and the carboxylate group than that of Cd^2+^ ion, which causes a larger coordination energy of Cu^2+^ with the oxygen-containing functional groups than that of Cd^2+^ ions. So the penetration of Cd^2+^ is greater than that of Cu^2+^. In terms of Cu^2+^ and Fe^3+^, due to the smaller size and higher charges of Fe^3+^ ions, the average distance between Fe^3+^ and the oxygen-containing functional groups is smaller than that of Cu^2+^, leading to the larger coordination energy of Fe^3+^ than that of Cu^2+^. Therefore, the penetration of Cu^2+^ is greater than Fe^3+^.

In terms of H^+^ ions in aqueous solutions, the H^+^ ions will be attracted by the hydrophilic sites in G-O and propagate through the hydrogen-bonding networks along the water layers in between the G-O sheets rapidly, which will result in an anomalous much faster transport of H^+^ than other cations (11). (More detailed discussions can be found in Ref. 3 and 12).

During the penetrations of CuCl_2_ and CuSO_4_, CdCl_2_ and CdSO_4_, it reveals in **Figure S5** that the concentrations of Cu^2+^ and Cd^2+^ in the cases of CuCl_2_ and CdCl_2_ are larger than those in the cases of CuSO_4_ and CdSO_4_, indicating that the penetrations of cations through G-O membranes are strongly dependent on the counter anions. Because of the higher charges of SO_4_^2-^ ions, the electrostatic drags from SO_4_^2-^ ions are stronger than those from Cl^-^ ions, leading to slower penetrations of cations in the cases of CuSO_4_ and CdSO_4_ compared to the cases of CuCl_2_ and CdCl_2_.

Based on the different interactions of cations with the *sp^2^* and *sp^3^* clusters of G-O sheets, the G-O membranes display efficient selectivity towards different cations, indicating that G-O membranes have potential applications in ion separations and waste water purification.

## Figure S6. pH variations of the filtrates in drains of selected salt solutions during the penetration process.

## Detailed principle for the generation of the trans-membrane electrical potential Δ*V*_DS_ across G-O membranes (illustrated in Figure 3a).

After membrane separation, the ions in sources and drains should possess different chemical potentials *μ_i_*, which could be obtained by the following equation:

*μ_i_* = *μ_i_^0+^* + *RT* ln*a_i_* = *μ_i_^0+^* + *RT* ln (*m_i_/m^0^*) + *RT* ln*γ_i_* (1)

In this equation, *μ_i_^0+^* is the chemical potential at unit activity, *R* is the gas constant, *T* is the temperature, *a*_i_ is the activity of ions (it can be obtained from *a*_i_ = *γ*_i_ *m*_i_*/m_0_*), *m_i_* is the molar mass concentration, *m^0^* is the normal molar mass concentration and *γ_i_* is the activity coefficient. In this case, ions should be spurred by a diffusion driving force that advances them to the drains, as illustrated in Equation (2):

*J_i_* = -*D_i_c_i_/RT* **grad** *μ_i_* (2)

*J_i_* is the amount of substance diffusing through per unit area per second, *D_i_* is the diffusivity and *c_i_* is the concentration. If the activity coefficients *γ_i_* for sources and drains were equal to each other, Equation (2) could be simplified to afford Fick’s first law:

*J_i_* = -*D_i_* **grad** *c_i_* (3)

For ions passing through G-O membranes, they should possess different diffusivities *D_i_*, presumably due to the different interactions between ions and G-O nano-capillaries, resulting in difference on ionic mobility, as described in the Nernst-Einstein equation:

*D_i_* = *u_i_k_B_T* (4)

In this equation, *u_i_* is the ion mobility and *k_B_* is the Boltzmann constant. Consequently, such mobility difference could lead to the presence of an excess amount of cations or anions in both drains and sources, which in turn should produce a trans-membrane electric potential Δ*V*_DS_. Under the isothermal and isobaric conditions, for each mol of reactant, the maximum electrical work should be equal to the molar free energy change:

Δ_r_ *G* = -*nF*Δ*V*_DS_ (5)

where Δ_r_ *G* is the molar free energy change, *n* is the number of charges transferred, *F* is the Faraday constant, *F* *=* *N*_A_*e*_0_, where *N*_A_ is the Avogadro constant. Theoretically, one could calculate the value of Δ*V*_DS_ from Δ*V*_DS_ = -Δ_r_ *G*/*nF*.

In addition, the substantial difference observed in the Δ*V*_DS_ values of different chlorides could be originated from the mobility (*u_i_*) difference of various ions.

*u_i_* = *z_i_e_0_/6πηr_i_* (6)

Theoretically, the mobility of an ion could be obtained from Equation (6), in which *z_i_e_0_* is the charge of the ion, *η* is the viscosity of medium, and *r_i_* is the radius of the ion. During the course of ion transporting through G-O membranes, a greater *u_i_* difference for either cations or anions often leads to a larger Δ*V*_DS_.

## Figure S7. Voltage generations across sources and drains Δ*V*_DS_ based on chlorides and sulphates with the same cations.

**(a)** 0.1 mol/L MgCl_2_ and MgSO_4_ solutions, **(b)** 0.1 mol/L CdCl_2_ and CdSO_4_ solutions. The solid lines are guides to eyes.

It reveals that the voltage generations across drains and sources for chlorides are generally larger than those for sulphates with the same cations, indicating that the counter anions affect the voltage generations significantly. According to Eq. (6), *u_i_* = *z_i_e_0_/6πηr_i_*, the mobility of SO_4_^2-^ is larger than that of Cl^-^ due to the higher charge and comparable radius of SO_4_^2-^ relative to Cl^-^. This effect results in the smaller differences in mobilities of SO_4_^2-^ and the corresponding cations, further leading to the smaller Δ*V*_DS_ generated by sulphates than by chlorides.

## Figure S8. Voltage generations across G-O membranes Δ*V*_GDS_ during the penetration process.

**(a)** Schematic diagram of the experiments. **(b)** Stable voltage generations across G-O membranes based on the penetrations of KCl, MgCl_2_, CaCl_2_, BaCl_2_, HCl and CdCl_2_ solutions for 3 h.

## The detailed principle for the generation of Donnan potential Δ*V*_GDS_ across G-O membranes (illustrated in Figure 3a).

In this study, the separation of sources and drains by G-O membranes has effectively prevented the free transportation of source ions, resulting in the establishment of an electrochemical dialysis equilibrium, which consequently should generate a Donnan potential.

For cations in source and drain solutions:

*μ*_+_^S^ *+ z*_+_*FV*_GS_ = *μ*_+_^D^ *+ z*_+_*FV*_GD_ (7)

For anions in source and drain solutions:

*μ*-^S^ *– z*-*FV*_GS_ = *μ*-^D^ *– z*-*FV*_GD_ (8)

In these two equations, *μ*_+_^S^, *μ*-^S^ and *μ*_+_^D^, *μ*-^D^ are the chemical potentials of cations and anions in source and drain solutions, respectively; *z*_+_ and *z*- are the valences of the cations and anions. Combing Equations (1), (7) and (8), we can calculate the voltages across G-O membranes Δ*V*_GDS_ by the following equation:

Δ*V*_GDS_ = *V*_GD_ *–* *V*_GS_ = *RT/F* ln (*a*_+_S/*a*_+_^D^) = *RT/F* ln (*a*-D/*a*-^S^) (9)

In this equation, *R* is the gas constant, *T* is the temperature, *F* is the Faraday constant, *F* *=* *N*_A_*e*_0_, where *N*_A_ is the Avogadro constant, *a*_+_^S^, *a*-^S^ and *a*_+_^D^, *a*-^D^ are the activities of cations and anions in source and drain solutions, respectively.

## Figure S9. Voltage generations Δ*V*_DS_ across G-O membranes with different diffusive areas.

The voltages were measured based on the penetrations of **(a)** KCl, and **(b)** MgCl_2_ solutions (0.1 mol/L). The solid lines are guides to eyes.

It reveals that shrinking the contact areas of G-O membranes to the sources and drains results in the increase of generated voltages across G-O membranes.

## Figure S10. Penetrations of selected salts and voltage generations across sources and drains when magnetic fields are applied.

**(a,b)** Schematic diagrams of the magnetic fields applied. **(c,d)** The generated voltages across sources and drains in the case of magnetic fields applied. The solid lines are guides to eyes.

The results indicate that when applying a magnetic field, the voltage generations across drains and sources display a significant enhancement, suggesting the transduction of magnetic energy to electricity.

## Figure S11. Voltage generations across sources and drains at different temperatures.

Voltage generations across sources and drains based on the penetrations of **(a)** 0.1 mol/L KCl solutions and **(b)** 0.1 mol/L MgCl_2_ solutions at 20 and 40 ^o^C, respectively. The solid lines are guides to eyes.

The results reveal that the voltage generations across drains and sources increase markedly with the increment of temperature, indicating the transduction of thermal energy to electricity. When the temperature was increased from 20 to 40 ^o^C, the enhanced thermal motions of ions should increase the ion flux through G-O membranes and weaken the attractions between cations and anions, which would result in a more effective separation of cations and anions by the G-O membranes, further leading to the increase of voltage generations. Theoretically, Δ*V*_DS_ can be described as Δ*V*_DS_ = -Δ_r_ *G*/*nF*, and Δ_r_ *G* can be obtained from the Gibbs-Helmholtz equation:

Δ_r_ *G* =Δ_r_ *H - T*Δ_r_ *S* (10)

In this equation, Δ_r_ *H* is the molar enthalpy change, and Δ_r_ *S* is the molar entropy change. Additionally, the functional relationship between Δ*V*_DS_ and *T* can be expressed as

(dΔ*V*_DS_ / d*T*) = Δ_r_ *S* / *nF*  (11)

Therefore, a significant entropy increase should be present in the ion transporting process of G-O membranes, in which a facile energy transduction from heat to electricity would be observed.

## Figure S12. Comparison of voltage generations across drains and sources under the conditions of heating and applying magnetic field.

The results exhibited in **Figure S12** reveal that both the Δ*V*_DS_ under the conditions of heating and applying magnetic field can be enhanced significantly compared to the pristine case. Surprisingly, when the ion trans-membrane transport process was conducted under the condition of simultaneous heating and magnetic field, the expected superimposed effect in the increase of Δ*V*_DS_ did not occur. On the contrary, the Δ*V*_DS_ values seemed to lie between the single cases, hinting that the enhancement of Δ*V*_DS_ in the cases of heating and applying magnetic field might follow adverse mechanisms. In detail, when the temperature was increased from 20 ^o^C to 40 ^o^C, the enhanced random thermal motion of ions would result in the significant increment of entropy during the ion transporting process of G-O membranes (Equation 11). In contrast, it can be deduced that when applying a magnetic field across the G-O membrane during the penetration process, the structure of the G-O nano-capillary network and the ionic migrations might become more ordered, which in turn would lead to the decrease of entropy. Following these adverse mechanisms, the superimposed increment of Δ*V*_DS_ did not occur under the conditions of both applying magnetic field and heating. (Detailed investigations on the magneto-induced modulation of the ion transport through G-O membranes are conducted somewhere else.)

## Table S1. Practical energy generations based on G-O membranes.

The generated voltages and currents are measured based on G-O membranes with different cross-section areas and solution volumes (in source and drain).

| Solutions  (1 mol/L/0.01 mol/L) | 5 mm in diameter of G-O membranes | | | 3 mm in diameter of G-O membranes | | |
| --- | --- | --- | --- | --- | --- | --- |
|  | Generated voltage (mV) | Generated  current (μA) | Power  (mW/m^2^) | Generated voltage (mV) | Generated  current (μA) | Power  (mW/m^2^) |
| KCl | 152.4 | 44.8 | 347.7 | 258.8 | 4.77 | 174.6 |
| CaCl_2_ | 80.4 | 24.4 | 99.9 | 231.4 | 4.46 | 146 |
| BaCl_2_ | 78.9 | 35.9 | 144.3 | 228.7 | 5.34 | 172.8 |
| HCl | 200.2 | 9.31 | 94.9 | 290.2 | 5.25 | 215.5 |
| FeCl_3_ | 57.6 | 44.3 | 130.0 | 64.0 | 26.4 | 239.0 |
| Solutions  (1 mol/L/0.01 mol/L) | 80 mL of solutions in sources/drains | | | 40 mL of solutions in sources/drains | | |
|  | Generated voltage (mV) | Generated  current (μA) | Power  (mW/m^2^) | Generated voltage (mV) | Generated  current (μA) | Power  (mW/m^2^) |
| KCl | 152.4 | 44.8 | 347.7 | 158.5 | 39.3 | 317.2 |
| CaCl_2_ | 80.4 | 24.4 | 99.9 | 76.9 | 31.4 | 122.9 |
| BaCl_2_ | 78.9 | 35.9 | 144.3 | 82.1 | 35.7 | 149.3 |

Practical energy generations by G-O membranes are investigated based on the selected salts as illustrated in **Table S1**. In order to reduce the essential resistance of the penetration system, 80 mL of selected salt solutions with a concentration of 1 mol/L and 0.01 mol/L are injected into the sources and drains respectively. The generated voltages and currents are measured across drains and sources based on G-O membranes with a diffusive area of 19.6 mm^2^ (5 mm in diameter) and 7.1 mm^2^ (3 mm in diameter) respectively. It reveals that decreasing the cross-section area of G-O membranes leads to the increase of generated voltages across drains and sources. At the same time, the generated currents decrease seriously. Though the calculated power densities (by *P*=*UI*/*A*, where *P* is the calculated power density, *U* is the generated voltage across drains and sources, *I* is the generated current and *A* is the diffusive area of G-O membranes) increase for most salts (except KCl), this is not an ideal route for practical applications considering the serious decrease of generated currents. However, as for KCl, increasing the membrane contacting area to source and drain leads to the significant enhancement of the calculated power density (from 174.6 to 347.7 mW/m^2^). In addition, the generated current is relative large for the case of G-O membranes with a cross-section area of 19.6 mm^2^, which shows promise for practical energy production application.

The electricity generation ability based on smaller volumes of sources and drains are further investigated. The vessels for sources and drains are reduced to half of their original volumes and 40 mL of 1 mol/L and 0.01 mol/L solutions are injected, respectively. It reveals that shrinking the penetration systems doesn’t lead to the significant change of the power densities, indicating that miniaturization of the penetration systems based on G-O membranes is a promising route for the design of practical electricity generation systems.

## Table S2. Energy generations based on G-O membranes under the conditions of heating and applying magnetic field.

| External conditions | KCl solutions (1 mol/L/0.01 mol/L)  5 mm in diameter of G-O membranes | | |
| --- | --- | --- | --- |
|  | Generated voltage (mV) | Generated current (μA) | Power density (W/m^2^) |
| Pristine | 152.4 | 44.8 | 0.35 |
| 50 mT | 248.1 | 82.2 | 1.04 |
| 40 ^o^C | 235.6 | 102.7 | 1.23 |
| 50 mT & 40 ^o^C | 196.1 | 103.5 | 1.03 |

It can be concluded from **Table S2** that when applying magnetic fields or heating, both the generated voltages and currents could be enhanced significantly. The calculated power densities could be improved from 0.35 W/m^2^ to 1.04 and 1.23 W/m^2^ (increased to 297% and 351%, respectively). However, when both applying magnetic field and heating, the generated voltage was lower than the single cases, suggesting the contrary mechanisms followed by the enhancement of voltage generations under the conditions of applying magnetic field and heating, as discussed in **Figure S12**. On the other hand, the generated current was improved significantly and the calculated power density was increased to 294% (from 0.35 W/m^2^ to 1.03 W/m^2^). These results indicated that magnetic and thermal energy could be efficiently converted to electricity. Furthermore, industrial magnetic leakage and waste heat could be collected for electricity reproduction using G-O membranes.

## Figure S13. Practical energy harvesting based on the penetrations of 1M/0.01M KCl through G-O membranes in series.

**(a)** Schematic diagrams of the electricity harvesting systems in series. **(b)** Series voltages, **(c)** essential resistances and **(d)** the calculated maximum output powers of the G-O membrane penetration systems in series. The solid lines are guides to eyes.


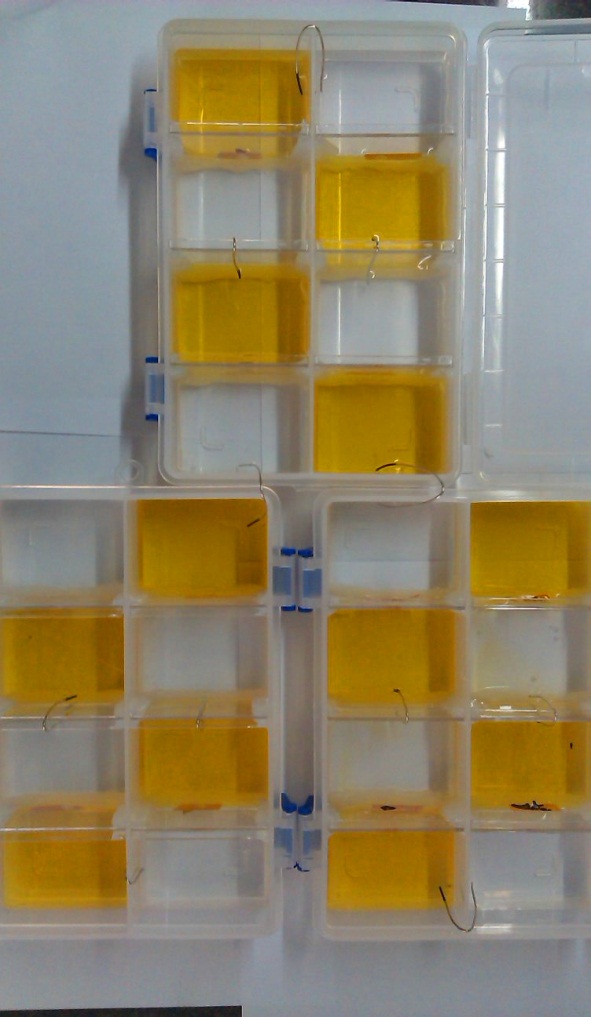


## Figure S14. Photograph of the penetrating system in series based on the penetration of FeCl_3_ through G-O membranes.

Several of the same penetrating systems based on the transport of KCl through G-O membranes were connected and the electricity generation performances of the series systems were studied preliminarily (**Figure S13a**). **Figure S14** exhibits an example of the series penetrating system based on the penetration of FeCl_3_, in which the sources and drains can be distinguished clearly by the different colors. It reveals that the generated voltage increases linearly with the number of systems in series (**Figure S13b**). However, due to the all-plastic nature of our penetration apparatus and poor connections between each system (by copper foils), the series resistances increase seriously (**Figure S13c**). In spite of this effect, the calculated power exhibits an increasing tendency when more than 2 of the penetration systems are in connection. These preliminary results indicate that it is promising to integrate the penetrating systems based on G-O membranes for enlarging the energy generation ability towards practical applications (**Figure 1c**).

Our previous results indicate that the hybridization of various salt solutions results in the improvement of voltages generated across the drains and sources (**Figure 3**), which inspires that industrial waste water, brine solutions, seawater and so on are ideal sources for energy generation based on G-O membranes. Meanwhile, ion separation can be achieved based on the selective penetration properties of G-O membranes. It is worth noting that the penetrating systems can be improved by using two conducting vessels (e.g. stainless steels) separated by an insulating plate which is sealed with G-O membranes. After integrating such penetrating systems together (**Figure 1c**), the series resistances can be significantly reduced while maintaining the original generated voltages. These results indicate that this new energy harvesting technology based on the miniaturization and integration of the G-O membrane-based penetrating systems is rather competitive or even exceeding the existing membrane-based energy generation processes such as PRO (pressure-retarded osmosis), RED (reverse electrodialysis) and MFC (microbial fuel-cell) (13). Moreover, it is shown in **Figure S13** that the connection of the penetrating systems results in the enhancement of generated power densities, indicating that integrated penetrating systems based on G-O membranes can improve the performance of energy generation markedly. During the ion transport process through G-O membranes, efficient transduction of magnetic, thermal and osmotic energy to electricity can be realized, which is absent in other energy harvesting systems. Also the penetrating systems based on G-O membranes don’t require bulky support layers, making the miniaturization and integration of the G-O membrane-based penetrating systems possible for practical electricity production applications. These excellent properties make G-O membranes rather competitive and promising compared to the existing membrane-based power generation processes such as PRO, RED and MFC (13).

## Figure S15. Transport properties based on commercial microfilters (220 nm).

**(a,b)** The conductivities and normalized conductivities of the drains. **(c)** Voltage generations across drains and sources. **(d)** Voltage generations across microfilters. The solid lines are guides to eyes.

## Figure S16. Transport properties based on commercial microfilters (220 nm).

**(a-b)** Voltage generations of KCl and MgCl_2_ when magnetic fields applied. **(c-d)** Voltage generations of KCl and MgCl_2_ under different temperatures. The solid lines are guides to eyes.

## Figure S17. Transport properties based on commercial microfilters (220 nm).

The penetrations of **(a)** KCl and **(b)** MgCl_2_ through microfilters with different contacting areas. The solid lines are guides to eyes.

In this study, the remarkable ion separation of G-O membranes should be inherently associated with their unique structural properties, which could be proved by control experiments using commercial microfilters (200 nm in aperture), as shown in **Figure S15-17**. Indeed, when ions were allowed to transport through the 200 nm microfilters, the conductivity of the drain solutions varied linearly with time (**Figure S15a**). For a dilute aqueous solution, the conductivity relates to the inherent properties of solutes, such as charge, mass, and size, and is approximately proportional to its concentration. Thus the normalized conductivity (calculated by dividing the conductivity of a 0.1 mol/L source solution) can be utilized to express the concentration change of drains, as demonstrated in **Figure S15**. As expected, microfilters did not exhibit selectivity for different ions in the penetration experiments. Subsequently, the Δ*V*_DS_ and Δ*V*_MDS_ in microfilters were also measured, which were similar to the ones obtained from G-O membranes, indicating that both materials followed the same confinement mechanism to generate voltage, as shown in **Figure S15c,d**. However, no significant change was observed in the Δ*V*_DS_ values when a similar weak magnetic field was employed, as shown in **Figure S16a,b**. This result unarguably demonstrated that G-O membranes could readily undergo energy transduction to convert magnetism to electricity during the course of ion transportation. When the temperature was increased from 20 to 40 ^o^C (**Figure S16c,d**), a much smaller enhancement of Δ*V*_DS_ was evident in the microfilter system, suggesting that a more efficient conversion from heat to electricity should be present in G-O membranes. Finally, the potential differences Δ*V*_DS_ across microfilters with different diffusive areas have been measured and the results were plotted in **Figure S17**. Notably, reducing the diffusive area of microfilters did not dramatically increase Δ*V*_DS_, hinting inefficient confinement and ion separations in such material. Based on these results, we have concluded that G-O membranes should afford superior energy conversion and ion separation, proving their advantageousness over semi-permeable membranes.

## Figure S18. Synchronous ion separation and energy generation based on G-O membranes.

Ion separation and energy generation processes of (**a),** MgCl_2_-MgSO_4_, (**b),** MgCl_2_-CuCl_2_, (**c),** MgCl_2_-FeCl_3_, (**d),** MgCl_2_-CuSO_4_, (**e),** NaCl-MgCl_2_ and (**f),** NaCl-FeCl_3_ hybrid solutions through G-O membranes. The insets quantify the concentrations of cations and anions of the drains after 3 h of penetration. The solid lines are guides to eyes.

Using hybrid sources in G-O membranes, we were able to achieve an ion separation process accompanied with synchronous energy generation, as shown in **Figure S18**. Particularly, the Δ*V*_DS_ generated from hybrid sources were larger than the ones from single-component sources. In terms of ion separation, we have found that Cl^-^ could be effectively separated from SO_4_^2-^, presumably because SO_4_^2-^ anions possess higher charges, leading to a stronger repulsion from G-O nano-capillaries (**Figures S18a and S18d**). In the case of hybrid cations, their permeability in G-O membranes was in the order of Na^+^ > Mg^2+^ > Fe^3+^, presumably because highly charged cations could afford stronger attractions towards the negatively charged G-O nano-capillaries (**Figures S18c, S18e and S18f**). Using single-component sources, Mg^2+^ and Cu^2+^ have been allowed to penetrate through G-O membranes, and their concentrations in the drain solutions were subsequently determined by emission spectroscopy analysis. These data were summarized in **Figure S5**. Notably, the concentration of Mg^2+^ was significantly higher than Cu^2+^ in drains. To the contrast, the concentration of Cu^2+^ was higher than Mg^2+^ in drains while using hybrid sources, as shown in **Figures S18b and S18d**. Our previous AES analysis showed that the concentrations of Mg^2+^ and C distributed symmetrically along the thickness of G-O membranes, indicating that Mg^2+^ ions have intercalated into the interlayer space of G-O laminates to form cation layers^3^. Surprisingly, it appears that the concentration of Cu^2+^ distributed randomly along the depth, indicating that Cu^2+^ ions should be captured by G-O sheets in a haphazard manner^12^. These results indicated that a stronger interaction was present between Cu^2+^ and G-O membranes, compared with Mg^2+^. When hybrid cations were allowed to penetrate through G-O membranes, Mg^2+^ layers along the G-O nano-capillaries should be first formed, which would induce a strong repulsion force and shielding effect towards the coordination of Cu^2+^ with the *sp^3^* matrix of G-O sheets, enabling them to move faster than Mg^2+^ ions, as illustrated in **Figures S18b and S18d**.

## References

1. Stankovich, S. et al. Synthesis of graphene-based nanosheets via chemical reduction of exfoliated graphite oxide. *Carbon* **45**, 1558-1565 (2007).
2. Gu, W. et al. Graphene sheets from worm-like exfoliated graphite. *J. Mater. Chem.* **19**, 3367-3369 (2009).
3. Sun, P. et al. Selective trans-membrane transport of alkali and alkaline earth cations through graphene oxide membranes based on cation-π interaction. *ACS Nano* **8**, 850-859 (2014).
4. Kumpf, R. A. & Dougherty, D. A. A mechanism for ion selectivity in potassium channels: computational studies of cation-pi interactions. *Science* **261**, 1708-1710 (1993).
5. Rao, J. S., Zipse, H. & Sastry, G. N. Explicit solvent effect on cation-*π* interactions: A first principle investigation. *J. Phys. Chem. B* **113***,* 7225-7236 (2009).
6. Cabarcos, O. M., Weinheimer, C. J. & Lisy, J. M. Size selectivity by cation–π interactions: Solvation of K^+^ and Na^+^ by benzene and water. *J. Chem. Phys.* **110**, 8429-8435 (1999).
7. Rodriguez-Cruz, S. E. & Williams, E. R. Gas-phase reactions of hydrated alkaline earth metal ions, M^2+^ (H_2_O)*_n_* (M = Mg, Ca, Sr, Ba and *n* = 4–7), with benzene. *J. Am. Soc. Mass Spectrom.* **12**, 250-257 (2001).
8. Rulı´sˇek, L. & Havlas, Z. Theoretical studies of metal ion selectivity. 1. DFT Calculations of Interaction Energies of Amino Acid Side Chains with Selected Transition Metal Ions (Co^2+^, Ni^2+^, Cu^2+^, Zn^2+^, Cd^2+^, and Hg^2+^). *J. Am. Chem. Soc.* **122**, 10428-10439 (2000).
9. Marino, T., Toscano, M., Russo, N. & Grand, A. Structural and electronic characterization of the complexes obtained by the interaction between bare and hydrated first-row transition-metal ions (Mn^2+^, Fe^2+^, Co^2+^, Ni^2+^, Cu^2+^, Zn^2+^) and Glycine. *J. Phys. Chem. B* **110**, 24666-24673 (2006).
10. Carrell, C. J., Carrell, H. L., Erlebacher, J. & Glusker, J. P. Structural aspects of metal ion-carboxylate interactions. *J. Am. Chem. Soc.* **110**, 8651-8656 (1988).
11. Karim, M. R. et al. Graphene oxide nanosheet with high proton conductivity. *J. Am. Chem. Soc.* **135**, 8097-8100 (2013).
12. Sun, P. et al. Selective ion penetration of graphene oxide membranes. *ACS Nano* **7**, 428-437 (2013).
13. Logan, B. E. & Elimelech, M. Membrane-based processes for sustainable power generation using water. *Nature* **488**, 313-319 (2012).
